# Blood Immune Cell Alterations in Patients with Hypertensive Left Ventricular Hypertrophy and Heart Failure with Preserved Ejection Fraction

**DOI:** 10.3390/jcdd10070310

**Published:** 2023-07-20

**Authors:** Artem Ovchinnikov, Anastasiya Filatova, Alexandra Potekhina, Tatiana Arefieva, Anna Gvozdeva, Fail Ageev, Evgeny Belyavskiy

**Affiliations:** 1 Laboratory of Myocardial Fibrosis and Heart Failure with Preserved Ejection Fraction, Institute of Clinical Cardiology, National Medical Research Center of Cardiology Named after Academician E.I. Chazov, 121552 Moscow, Russia; anastasia.m088@yandex.ru (A.F.); potehina@gmail.com (A.P.); gvozdevaannalech@gmail.com (A.G.); 2 Department of Clinical Functional Diagnostics, A.I. Yevdokimov Moscow State University of Medicine and Dentistry, 127473 Moscow, Russia; 3 Laboratory of Cell Immunology, Institute of Experimental Cardiology, National Medical Research Center of Cardiology Named after Academician E.I. Chazov, 121552 Moscow, Russia; areftan2@gmail.com; 4 Out-Patient Department, Institute of Clinical Cardiology, National Medical Research Center of Cardiology Named after Academician E.I. Chazov, 121552 Moscow, Russia; ftageev@gmail.com; 5 MVZ des Deutschen Herzzentrums der Charité, 13353 Berlin, Germany; evgeny.belyavskiy@dhzc-charite.de

**Keywords:** left ventricular hypertrophy, type 2 diabetes mellitus, diastolic dysfunction, heart failure with preserved ejection fraction, T-cells, monocytes

## Abstract

(1) Background: Chronic inflammation and fibrosis are key players in cardiac remodeling associated with left ventricular hypertrophy (LVH) and heart failure with a preserved ejection fraction (HFpEF). Monocytes and T-helpers (Th) are involved in both pro-inflammatory and fibrotic processes, while regulatory T-cells (Treg) could be considered to suppress chronic inflammation in the hypertrophied myocardium. We aimed to estimate the relationship between the frequencies of circulating CD4^+^ T-cell and monocyte subpopulations and the variables of left ventricular (LV) diastolic function in patients with LVH depending on the presence of HFpEF. (2) Methods: We enrolled 57 patients with asymptomatic hypertensive LVH (*n* = 21), or LVH associated with HFpEF (*n* = 36). A clinical assessment and echocardiographs were analyzed. CD4^+^ Treg, activated Th (Th-act), and monocyte (classical, intermediate, and non-classical) subpopulations were evaluated via direct immunofluorescence and flow cytometry. (3) Results: Patients with HFpEF had a lower Treg/Th-act ratio (*p* = 0.001). Though asymptomatic patients and patients with HFpEF were comparable in terms of both the total monocyte number and monocyte subsets, there were moderate correlations between intermediate monocyte count and conventional and novel echocardiographic variables of LV diastolic dysfunction in patients with HFpEF. (4) Conclusions: In patients with LVH, the clinical deterioration (transition to HFpEF) and progression of LV diastolic dysfunction are probably associated with T-cell disbalance and an increase in intermediate monocyte counts.

## 1. Introduction

Approximately half of patients with heart failure have a normal ejection fraction (HFpEF), and the prevalence of this heart failure phenotype is constantly increasing [1]. The 5-year mortality rate in HFpEF is 50%, while it reaches 75% in patients hospitalized due to an exacerbation of heart failure [2]. To date, the wide range of pharmacotherapies improving the prognosis of heart failure with a low ejection fraction (HFrEF)—renin-angiotensin system blockers, beta-blockers, and aldosterone antagonists—have shown a minimal impact on the outcomes, exercise capacity and quality of life in HFpEF. This is probably explained by the difference in pathogenetic mechanisms: cardiomyocyte death in HFrEF versus chronic myocardial microvascular inflammation in HFpEF [3].

According to the novel HFpEF paradigm, proinflammatory comorbidities such as obesity, arterial hypertension, type 2 diabetes, chronic kidney disease, chronic obstructive pulmonary disease, and anemia trigger a low-grade systemic inflammatory status and coronary microvascular endothelial dysfunction with subsequent cardiomyocyte hypertrophy, myocardial infiltration with activated leukocytes and reactive fibrosis [4]. A growing body of evidence supports the causative role of a comorbidity-driven, systemic pro-inflammatory state in HFpEF [5].

Left ventricular hypertrophy (LVH) is a common structural cardiac alteration in HFpEF. The most prevalent cardiovascular condition associated with LVH is arterial hypertension. Despite the compensatory nature of LVH, which is aimed at adapting to higher demands for LV work, subsequent extracellular matrix deposition occurs with progressive LV diastolic dysfunction (LVDD) and a reduction in capillary density [6]. In all these processes, innate and acquired immunity reactions may play principal roles, where the key event is the migration of monocytes from the bloodstream into myocardial interstitial space with subsequent transformations into macrophages [7]. In the inflammatory process, macrophages and CD4^+^ T-lymphocytes actively interact with each other. Macrophages act as antigen-presenting cells for T-cells, express costimulatory molecules and produce cytokines (interleukin-12, etc.) that activate T-lymphocytes. In turn, T-cells activated via cytokines (interferon-γ, and interleukines-4, -5, and -13) promote macrophage activation and differentiation [8]. In rodent models of LVH caused by pressure overload, increased monocyte/macrophage recruitment with myocardial infiltration has been associated with exaggerated fibrosis, and diastolic dysfunction has been demonstrated to occur in the early stages of LVH [9,10]. Tissues collected via cardiac biopsy from patients with HFpEF revealed a significant accumulation of activated macrophages producing transforming growth factor-beta, fibroblast activation and excessive myocardial collagen deposition [10,11]. Similarly, a significant increase in the content of pro-inflammatory monocytes was detected in the blood of patients with hypertensive HFpEF [12]. Experimental and clinical studies have shown that myocardial fibrosis is also related to CD4^+^ T-cell infiltration [13,14]. However, despite research efforts to date, the identification of the predominant immune cell types responsible for the proinflammatory myocardial changes in LVH and HFpEF has not yet been successfully carried out, particularly in humans.

The low-grade chronic inflammation specific to HFpEF can be further exacerbated by type 2 diabetes mellitus (T2DM). Increased myocardial extracellular matrix accumulation in T2DM plays a critical role in the worsening of LVDD [15]. Considering the large amount of evidence suggesting a role for macrophages and T-cells in fibroblast activation in rodent models with pressure overload [7], it is plausible to hypothesize that immune cell recruitment and activation may be involved in diabetic cardiomyopathy. Nevertheless, to date, limited information is available on the potential role of T-cells and monocytes in diabetic HFpEF. In the present cross-sectional study, we aimed to estimate the relationship between CD4^+^ T-cell and monocyte blood frequencies and the variables of LV diastolic and systolic function in patients with LVH depending on the presence of HFpEF and/or T2DM.

## 2. Materials and Methods

### 2.1. Study Population

This single-center study was cross-sectional and a part of a prospective randomized trial evaluating the efficacy of sacubitril/valsartan in patients with HFpEF and LVH (NCT03928158). The study population consisted of 57 Caucasian hypertensive subjects aged ≥ 40 years with asymptomatic concentric LVH (*n* = 21), or LVH associated with HFpEF (*n* = 36). Asymptomatic LVH was evidenced by the absence of any exercise limitations and LV diastolic dysfunction (DD) of grade I. HFpEF was diagnosed in accordance with the current recommendations as follows: symptoms and/or signs of HF, preserved LV ejection fraction (≥50%), and an elevated LV filling pressure verified during rest or during exercise via echocardiography [16].

Those patients with alternative causes of LVH, asymmetrical or eccentric LVH, secondary hypertension, myocardial ischemia during stress echocardiography, chronic atrial flutter/fibrillation, LV dilatation (a LV end-diastolic dimension of ≥5.9 cm in men and ≥5.3 cm in women), significant left-sided structural valve disease, infiltrative or inflammatory myocardial diseases, or noncardiac conditions precluding participation were excluded. This study was approved by the Ethics Committee of the Institute of Clinical Cardiology and complied with the Declaration of Helsinki. All patients provided written informed consent. Echocardiography (during rest and during exercise), a 6 min walk test distance (6MWD) procedure, blood analyses for N-terminal pro-B-type natriuretic peptide (NT-proBNP), and a determination of immune and inflammatory cell content were performed.

### 2.2. Echocardiography

An echocardiographic assessment was performed using a Vivid E95 ultrasound system (GE Healthcare, Horton, Norway). Wall thickness, chamber volumes, and LV ejection fraction were determined in accordance with the current guidelines [17]. LVH was defined as a LV mass index of >115 g/m^2^ in men and >95 g/m^2^ in women. The relative wall thickness (RWT) was defined as (septal wall thickness + posterior wall thickness)/LV end-diastolic dimension with a further categorization of an increase in the LV mass index as either concentric (RWT > 0.42) or eccentric (RWT ≤ 0.42) hypertrophy [17]. LV diastolic function was assessed by measuring the mitral inflow velocities (E, A), averaged mitral annulus relaxation velocity (mitral e′), and mitral E/e′ ratio. The severity of LVDD was determined in accordance with the 2016 ASE criteria for the grading of LV diastolic dysfunction [16]. An elevated LV filling pressure during rest was verified if LV diastolic dysfunction of grade II–III was revealed, and that during exercise (during supine bicycle exercise) was verified if an exercise-induced elevation of E/e′ (average E/e′ > 14) and a tricuspid regurgitation velocity (>2.8 m/s) were observed [16].

Right heart assessment included right ventricle (RV) size, systolic function (M-mode tricuspid annular plane systolic excursion (TAPSE)), and diastolic function (pulsed Doppler of tricuspid inflow; tissue Doppler of lateral tricuspid annulus (e′ and E/e′ ratio)) [17]. Pulmonary artery systolic pressure (PASP) was calculated as a sum of peak tricuspid regurgitation and right atrial pressure was estimated via the inferior vena cava’s size and its collapse. For measuring the acceleration time of the RV outflow velocity curve (AcT_RVOT_), a Doppler sample volume was placed in the center of the RVOT proximally to the pulmonic valve. AcT_RVOT_ is normally greater than 105 ms and shortens in proportion to elevations in pulmonary vascular resistance.

**Figure 1:**
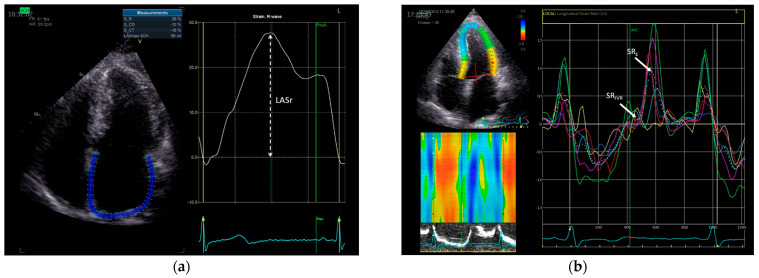
An example of (**a**) LA reservoir strain (LASr), and (**b**) LV global longitudinal strain during early diastole (SR_E_) and during the isovolumic relaxation period (SR_IVR_) in a patient with HFpEF. LA strain was determined as the average value of the longitudinal positive strain peak under LA relaxation from all six segments of the LA in the apical 4-chamber view, using the onset of the QRS as the referent point. The white dotted curve represents the average value of LA strain from all analyzed LA segments. SR_E_ and SR_IVR_ were determined as the average values of the longitudinal positive strain rate peak during LV early diastole and isovolumic relaxation period, respectively, from all LV segments in the apical 4-chamber view. Different color lines represent strain rate curves of individual LV segments. The white dotted curve corresponds to the average value of LV strain rate from all six analyzed LV segments. LA, left atrial; LV, left ventricular.

Deformation analysis using two-dimensional speckle-tracking echocardiography was performed offline using the dedicated ultrasound software package (Echo-Pac version 203, GE Healthcare) at frame rates of 50–80 frames/s. LA strain was calculated as the average strain in six segments of the left atrium (LA) in an apical four-chamber view to calculate LA global longitudinal reservoir strain (LAS_r_) (Figure 1) [18]. LV global longitudinal strain during systole (LV GLS), as well as global strain rates during early diastole (SR_E_) and during the isovolumic relaxation period (SR_IVR_) were measured from the apical views [19]. All echocardiographic measures represent the mean of ≥3 beats.

**Figure 2:**
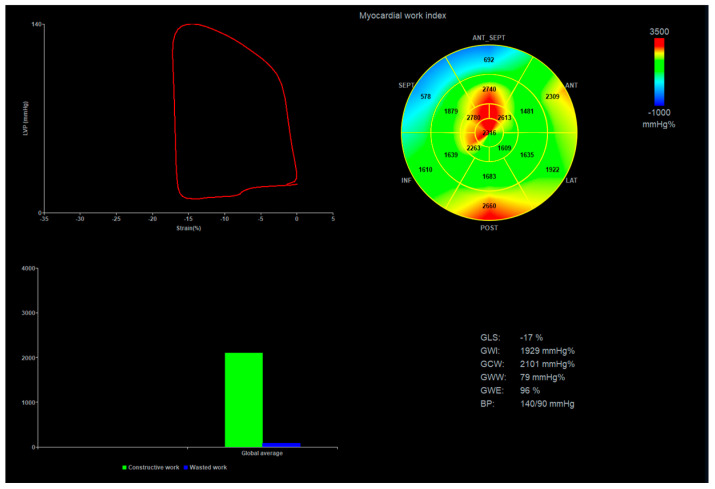
An example of myocardial work analysis in a patient with HFpEF. The LV pressure-strain loop is presented in a 17-segment myocardial work bull’s-eye plot; the proportion of constructive (green) and wasted work (blue) and calculations are presented. BP, blood pressure; GWE, global myocardial work efficiency; GWI, global myocardial work index; GLS, global longitudinal strain; GCW, global constructive work; GWW, global wasted work.

Myocardial work was assessed via the method introduced by Russell et al. [20] and was analyzed using the dedicated ultrasound software package (Echo-Pac version 203, GE Healthcare) after the calculations of LV GLS and peak noninvasive systolic blood pressure were inputted. Pressure–strain loops were synchronized with the opening and closing times of the aortic and mitral valves. Myocardial work was quantified by calculating the rate of regional shortening by differentiating strain tracing and multiplying the result by the instantaneous LV pressure integrated over time (Figure 2). The following parameters were obtained: the global work index (GWI; mmHg %), representing the area within the LV pressure–strain loop; the global constructive work (GCW; mmHg %), representing the LV work generated via the shortening of the myocardium during systole and its lengthening under isovolumetric relaxation; the global wasted work (GWW; mm Hg %), representing the amount of ineffective energy with LV lengthening during systole and shortening under isovolumetric relaxation; and the global work efficiency (GWE; %), which was calculated as GCW/(GCW + GWW).

### 2.3. Diastolic Stress Test (DST)

Patients exercised supine cycle ergometry at 60 rpm starting with a 3 min period of a low-level 25 W workload followed by 25 W increments in 3 min stages to the maximal tolerated levels or until the patient developed limiting symptoms. During the test, the changes in LV filling pressures (the mitral E/e′ ratio and TRV), and LV systolic (EF, GLS, average systolic mitral annulus tissue Doppler velocity (s′)), diastolic (mitral e′), and LA (LASr) functions during rest and at the peak of exercise were analyzed. An elevated LV filling pressure during exercise was verified if exercise-induced elevations in E/e′ (average E/e′ > 14) and TRV (>2.8 m/s) were observed [16].

### 2.4. NT-proBNP

The plasma level of the myocardial stress marker N-terminal pro–brain natriuretic peptide (NTproBNP) was measured via an automated electrochemiluminescence immunoassay (Roche Diagnostics, Mannheim, Germany). The detection limit of the NTproBNP assay was 5 pg/mL.

### 2.5. Lymphocyte and Monocyte Immunophenotyping

Whole blood was collected in sodium citrate anticoagulated vacutainer tubes. The samples were processed within 2 h after being collected. For surface antigen staining, the following antibodies and reagents were used: CD4-FITC, CD14-PE, CD16-FITC, CD25-PE, CD127-PC5, CD45-APC, and lysing solution (Beckman Coulter, Becton Dickinson Immunocytometry Systems, eBioscience, San Diego, CA, USA). The samples were analyzed with FACS Calibur and a FACS Canto flow cytometer (BD Immunocytometry Systems, San Jose, CA, USA). Lymphocytes and monocytes were gated according to the light scattering parameters and CD45 expression pattern. Regulatory T-cells (Treg) were identified as CD4^+^CD25highCD127low, and activated T-helper cells (Th-act) were identified as as CD4^+^CD25lowCD127high as described earlier [21]. Monocytes were identified as classical (CD14^++^CD16^–^), intermediate (CD14^++^CD16^+^) and non-classical (CD14^+^CD16^++^) in accordance with the routinely used protocol [22].

### 2.6. Statistical Analysis

According to our previous study, the difference in absolute values of Treg in 75 patients with and without coronary atherosclerosis was used to estimate the sample size needed to achieve adequate statistical power for the current study [23]. Based on a comparison of two means between groups, the difference of 14 × 10^3^/mL and a standard deviation of 15 and 17 × 10^3^/mL in patients with coronary atherosclerosis and in controls, respectively, at an α of 0.05 (two sided), a sample size of 25 patients per group was required to achieve a power of 80%.

Statistical analysis was performed using standard software (MedCalc, version 19.5.3). Data are presented as the median (interquartile range); categorical variables are reported as the numbers and percentages of observations. The differences in parameters between groups were tested using the Mann–Whitney U test, and the χ^2^ test for qualitative data. We evaluated the differences between the three groups in terms of quantitative variables using the Kruksal–Wallis ANOVA rank analysis of variance. The correlation between continuously distributed variables was tested through univariate regression analysis. For sensitivity analysis, receiver operating characteristic (ROC) analysis was applied. A value of *p* < 0.05 was considered statistically significant.

## 3. Results

### 3.1. Patient Characteristics

A comparison of the patient’s groups is shown in Table 1. Asymptomatic patients were comparable to patients with HFpEF in terms of age, body mass index and comorbidities; however, most of the following comorbidities were more prevalent in patients with HFpEF: type 2 diabetes mellitus, at 44% vs. 24%; paroxysmal atrial fibrillation, at 47% vs. 33%; obesity, at 64% vs. 48%; chronic kidney disease, at 25% vs. 19%. There was a higher rate of use of diuretics and RAAS blockers in patients with HFpEF.

Although all study participants had concentric LVH (an inclusion criterion), patients with HFpEF showed a higher LV mass index compared to asymptomatic patients (*p* = 0.03). Patients with HFpEF demonstrated clear differences in variables associated with LV diastolic dysfunction/filling pressure, including a higher LA volume, E/e′ ratio, and pulmonary artery systolic pressure and lower e′ velocity and LASr compared to asymptomatic patients. As a result, NT-proBNP, a marker of LV wall stress, was higher in HFpEF patients (*p* < 0.001).

**Figure 3:**
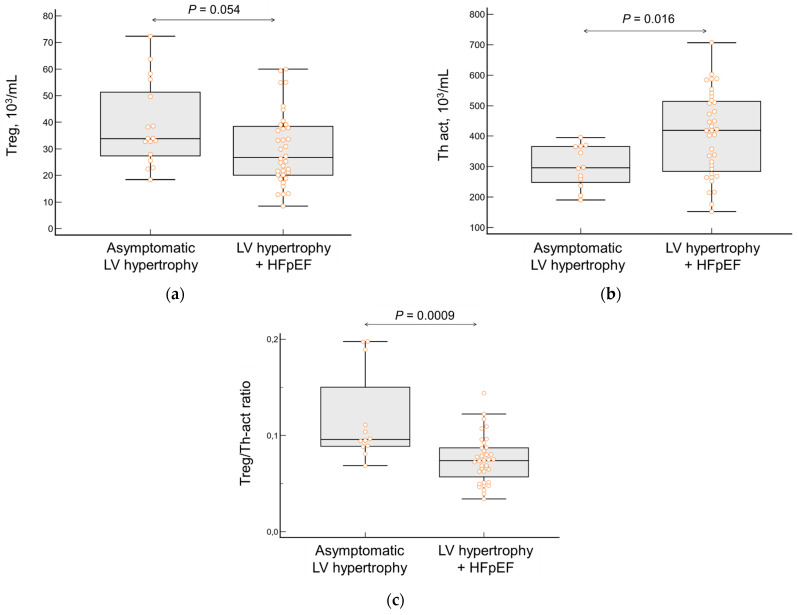
The total number of (**a**) regulatory T-cells (Treg), (**b**) activated T-helper cells (Th-act), and (**c**) their ratio in patients with asymptomatic LVH vs. those with HFpEF. Orange dots correspond to the values of the variable in individual patients.

**Figure 4:**
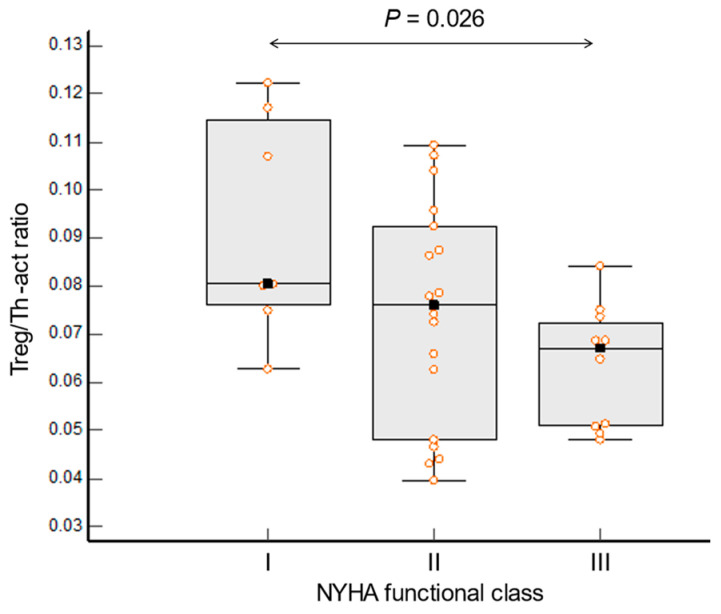
Ratio of regulatory T-cells to activated T-helper cells (Treg/Th-act) and functional severity in patients with HFpEF. Results were stratified by the severity of heart failure expressed by the New York Heart Association (NYHA) functional class and were compared via the Kruksal–Wallis ANOVA rank analysis of variance. Orange dots correspond to the values of the variable in individual patients.

No difference was found between asymptomatic and HFpEF patients in the total number of leukocytes, lymphocytes, CD4^+^ T-cells, and monocytes, as well as in the number of monocyte subpopulations (classical, non-classical, or intermediate). Patients with HFpEF had a significantly higher content of Th-act (*p* = 0.016), a tendency to have a lower content of Treg (*p* = 0.054), and, as a consequence, a lower Treg/Th-act ratio (*p* < 0.001, Figure 3a–c). In patients with HFpEF, this ratio decreased with NYHA functional class advancement (Figure 4).

The variables with statistical differences between asymptomatic and HFpEF groups were included in the receiver operating characteristic (ROC) analysis to determine their diagnostic accuracy in revealing HFpEF. The Treg/Th-act ratio was comparable to conventional LV diastolic parameters (E/e′ ratio, LA volume, PASP and NT-proBNP) and exceeded the LASr and LV mass in predicting HFpEF according to the ROC analysis (Table 2).

### 3.2. Immune Cell Correlates of Cardiac Function in HFpEF

**Figure 5:**
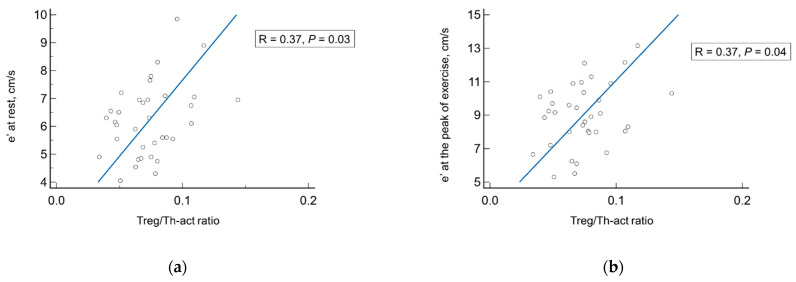

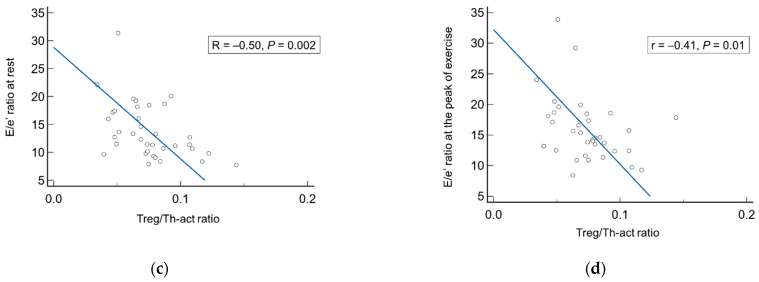
Correlations between echocardiographic parameters of LV diastolic function/filling pressure (e′ velocity and E/e′ ratio) both during rest (**a**,**c**) and at the peak of exercise (**b**,**d**), and Treg/Th-act ratio in patients with LVH and HFpEF. E, early inflow velocity; e′, averaged annulus relaxation velocity; Th-act, activated T-helper cells; Treg, regulatory T-cells. Black dots correspond to the values in individual patients.

Among patients with HFpEF, the Treg/Th-act ratio showed a significant association with several echocardiographic parameters of LVDD/filling pressure—E/e′ ratio and e′ velocity both during rest (r = −0.50 and 0.37, respectively) and at the peak of exercise (r = −0.41 and 0.37, respectively, Figure 5), and LAS_r_ (r = 0.34)—as well as the parameters of right cardiac chambers—inferior vena cava size (r = −0.34), and AcT_RVOT_ (r = 0.37, for all *p* < 0.05).

In patients with HFpEF, the total number of intermediate monocytes significantly correlated with a panel of echocardiographic variables reflecting various cardiac functions: LV diastolic function/filling pressure (E/e′ ratio, e′ velocity, SR_IVR_, SR_E_, pulmonary vein S/D ratio, and LASr), LV contractility (ejection fraction, GLS during rest and amplitude of exercise-induced GLS elevation, representing LV systolic reserve), myocardial work indexes (GWI, GWE, and GCW), right heart chamber function (TAPSE, indicating RV contractility, and tricuspid E/e′ ratio, indicating central venous pressure). In each case, higher numbers of intermediate monocytes corresponded to a poorer variant of echocardiographic findings (Table 3). Classical and non-classical monocytes correlated with fewer echocardiographic parameters, but the same pattern was observed; higher levels were associated with worse specific cardiac function (Table 3). Interestingly, none of the monocyte’s cellular subpopulations correlated with the severity of LV hypertrophy (LV mass index).

### 3.3. Immune Cell Values in Patients with HFpEF Depending on Diabetes Status

T2DM is a potent proinflammatory disease and plays a significant role in triggering and maintaining chronic inflammation in the myocardium in HFpEF [5]. We compared clinical, echocardiographic and immune cell parameters in patients with HFpEF and T2DM, and in patients with HFpEF without T2DM. Given the small number of diabetic patients among asymptomatic LVH patients (*n* = 5), we did not perform a similar comparison in the asymptomatic subgroup.

Patients with HFpEF and T2DM had more pronounced functional limitations (a higher NYHA functional class and shorter distance in the 6 min walking test), a higher body mass index compared to that of non-diabetic patients (in all cases *p* < 0.05). In addition, diabetic patients were characterized by significantly higher LV filling pressures (E/e′ ratio, *p* = 0.049), lower TAPSE values (*p* = 0.015) and trends toward worse LV reservoir function (lower LASr, *p* = 0.064) and higher NT-proBNP (*p* = 0.065) (Table 4).

**Figure 6:**
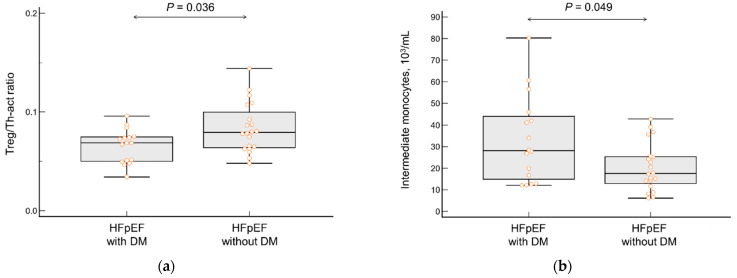
(**a**) The regulatory T-cell/activated T-helper cell ratio and (**b**) intermediate monocyte blood count in patients with HFpEF and type 2 diabetes mellitus (T2DM) vs. patients with HFpEF alone (**b**). Orange dots correspond to the values of the variable in individual patients.

Patients with T2DM had a significantly lower Treg/Th-act ratio (*p* = 0.036) compared to that of non-diabetic patients. These subgroups did not differ in total blood monocyte content or in the content of classical and non-classical monocyte subpopulations, but diabetic patients had significantly higher intermediate monocyte levels (*p* = 0.049; Table 4; Figure 6).

## 4. Discussion

The current study showed that in patients with hypertensive LVH, clinical deterioration (transition to HFpEF) is probably associated with CD4^+^ T-cell imbalance (a decrease in the Treg/Th-act ratio) but not with significant changes in monocyte count. However, both the Th-act/Treg ratio and intermediate monocyte levels were associated with LVDD/filling pressure. Thus, these changes may indicate the exacerbation of inflammatory processes within the hypertrophied myocardium and are consistent with the evolving paradigm of HFpEF as a chronic inflammatory condition associated with myocardial fibrosis [4].

The differences in immune cell levels between asymptomatic and HFpEF patients may be partially explained by the different pro-inflammatory comorbidity burdens in patients with HFpEF (Table 1). The reduction in the Treg/Th-act ratio in patients with HFpEF was predominantly due to an increase in Th-act levels and a tendency of levels of to Treg decrease. These data are consistent with a study by Lu M. et al. showing a Th17/Treg imbalance (increased Th17 cells and decreased Treg) in patients with heart failure and increased myocardial fibrosis via the expression of lysyl oxidase [24]. This enzyme catalyzes cross-links in collagen contributing to a left ventricular stiffness increase. Th17/Treg imbalance was also found in patients with cardiac inflammatory diseases such as acute coronary syndrome [25], and rheumatic heart disease [26].

CD4^+^ T-cells dominate in inflammation in the hypertrophied myocardium, acting as a ‘transmission link’ between chronic microvascular inflammation and myocardial fibrosis by secreting cytokines that direct M1/M2 macrophage differentiation [13]. In patients with non-ischemic HF, myocardial fibrosis was directly related to T-cell infiltration [14]. T-cell-deficient mice failed to develop aortic constriction-induced cardiac hypertrophy and fibrosis [14,27].

The main pool of regulatory cells mature in the thymus (natural Tregs). In the periphery, depending on the microenvironment, naive CD4^+^ T-cells differentiate into different Th effector populations and into inducible Tregs [28]. In pressure overload-induced HF, myocardial infiltration by effector CD4^+^ Th1 cells leads to the activation of cardiac fibroblasts with subsequent transformations into myofibroblasts and the expression of transforming growth factor-β [29]. Interferon-ɣ^+^ T-cells correlated with the NYHA functional class and serum brain natriuretic peptide levels in outpatients with heart failure [30]. Treg acts to resolve inflammation by secreting anti-inflammatory interleukin-10 [31] and inhibiting the activity of effector cells, including Th1 and macrophages [31,32]. In rodent models with angiotensin II infusion [33] or abdominal aortic constriction [24], the adoptive transfer of Treg reduced myocardial infiltration through macrophages and ameliorated cardiac hypertrophy and fibrosis. A decrease in the CCR10^+^ Treg subpopulation with increased immunosuppressive function has been shown in patients with hypertension [34]. The excess of Th-act that we have observed may indicate the increased readiness of the T-cell lineage to mount immune responses, including those in the myocardium [7].

Macrophages are key mediators of homeostasis in cardiac tissue. Early myocardial inflammatory events involve monocyte activation with extravasation and subsequent transformation into macrophages. In mouse models of LVDD, myocardial macrophage infiltration has been shown to be associated with fibrosis, suggesting that fibrosis could be prevented by suppressing inflammation [10,35,36]. Human HFpEF endomyocardial biopsies show a higher abundance of macrophages, predominantly of peripheral origin [10,11,37,38], and macrophages directly contribute to the development of fibrosis and LVDD [10,11].

In the present study, asymptomatic patients and patients with HFpEF did not differ in either total monocyte counts or monocyte subsets, although a significant increase in the content of both classical and non-classical monocytes has previously been reported in the blood of patients with HFpEF [12]. The absence of an increase in monocyte content but an increase in T-cell imbalance during the transition from asymptomatic LV hypertrophy to HFpEF may be due to the different modes of activation of these two immune processes. Monocyte/macrophage activation is dominant in early pressure overload and LVDD [9,12,39], while T-cell imbalance is anticipated at the advanced stages when the transition to HFpEF is established. In pressure-overloaded mice, the recruitment of monocyte-derived C-C chemokine receptor 2 macrophages to the myocardium precedes CD4^+^ T-cell infiltration [40,41].

In a cross-sectional study, asymptomatic hypertensive patients with mildly elevated levels of brain natriuretic peptide had increased levels of interleukin-6, tumor necrosis factor-α, and C-reactive protein, and an increased LV mass and LA volume [42]. Increased inflammatory markers were independently associated with asymptomatic LVDD in patients with arterial hypertension and metabolic syndrome [43]. Moreover, asymptomatic individuals with even slight evidence of low-grade vascular inflammation have been shown to be at an elevated risk of subsequent major cardiovascular events [44]. These data suggest that evidence of systemic and cardiac inflammation precedes that of symptomatic HF and indicates increased cardiovascular risk in hypertensive patients. All of our asymptomatic patients had LVH and LVDD, in which fibro-inflammatory features of the monocyte/macrophage system may have already been established. In a study by Glazeva N. et al., pro-inflammatory monocyte numbers were increased in both asymptomatic LVDD and HFpEF patients and correlated with LV function [12]. In the present study, both T-cell imbalance and intermediate monocytes reflected HFpEF severity, as evidenced by their significant correlations with many conventional and novel echocardiographic indices related to LV diastolic function/filling pressure, LA reservoir function, and LV and RV contractile functions. This may indicate an important involvement of immune processes in HFpEF pathophysiology, although the current study cannot prove a cause-and-effect relationship given its design.

In the present study, in contrast to intermediate monocytes, classical and non-classical monocytes were associated with a smaller number of echocardiographic parameters, probably indicating their involvement in a different focus of action. Classical monocytes with their subsequent preferential transformation into pro-inflammatory M1 macrophages are thought to be predominantly involved in reactions of acute inflammation, for example, in myocardial infarction, while intermediate and non-classical monocyte subsets are prone to transformation into reparative M2 macrophages and predominantly mediate reactions of chronic inflammation, for example, in atherosclerosis, rheumatoid arthritis, sarcoidosis, and inflammatory bowel disease [45]. In a study of Barisione C. et al., patients with stable HF and a reduced ejection fraction had increased levels of intermediate monocytes compared to those of healthy controls, and intermediate monocytes reflected disease severity [46]. Although to date there are no firm human biopsy data confirming the predominance of reparative (profibrotic) over proinflammatory macrophages in HFpEF, the numerous associations of intermediate monocytes with LV dysfunction we have identified may reflect the propensity of a monocyte phenotype to change into one of intermediate monocytes and then into exaggerated fibrosis. The control of macrophage activity may be a promising therapeutic strategy in HFpEF because it might minimize excessive LVH and fibrosis, and thereby prevent the progression of LV dysfunction [12,45].

Interest in the role of inflammation and immune disorders in the pathogenesis of HFpEF has increased significantly due to many comorbidities provoking and enhancing the systemic pro-inflammatory state. One of the most significant pro-inflammatory comorbidities is T2DM. To date, there is abundant evidence linking T2DM to systemic inflammation [47]. Systemic pro-inflammatory effects of T2DM are mainly promoted through the induction of the adhesion molecule and CC chemokine expression [48,49]. Approximately 45% of all patients with HFpEF have T2DM, and T2DM significantly increases morbidity and mortality in patients with HFpEF [50]. Diabetes-induced systemic inflammatory status predicts incident HFpEF, but not incident HFrEF [51].

From a pathophysiological point of view, T2DM exacerbates the course of HFpEF via several mechanisms: toxic intermediates and reactive oxygen species, accumulated due to insulin resistance [5], a reduction in nitric oxide bioavailability [52], and the secretion of proinflammatory cytokines by the myocardium and epicardium with the subsequent involvement of monocytes [53]. All these mechanisms worsen LVDD thorough increased myocardial extracellular matrix accumulation and the presence of stiffer cardiomyocytes [54,55,56]. Experimental studies have shown that multiple pro-inflammatory cascades are involved in diabetes-associated cardiac fibrosis [7]. In HFpEF trials, T2DM is represented by an elevated LV mass and LV filling pressure and reduced LV distensibility, as well as endothelial and coronary microvascular dysfunction [5].

In the present study, T2DM was associated with advanced HFpEF (more severe functional limitations and a higher LV filling pressure). In addition, patients with coexisting T2DM and HFpEF had more pronounced immune CD4^+^ T-cell imbalances (a lower Treg/Th-act ratio and lower Treg content) and significantly higher intermediate monocytes compared to patients with HFpEF alone. These changes appear to reflect an exacerbation of chronic inflammation in the myocardium. In obesity, adipose tissue expansion leads to the activation of pro-inflammatory macrophages and the secretion of cytokines, which plays an important role in the development of insulin resistance and T2DM [57]. Macrophages and T-cells also infiltrate the heart in diabetic cardiomyopathy, but their role in the pathogenesis of myocardial inflammation in coexisting HFpEF has not yet been addressed. Several lines of evidence support the role of monocytes/macrophages in diabetic cardiac fibrosis. The infiltration of the myocardium by monocytes has been consistently demonstrated in T2DM models [58,59], and hyperglycemia has induced cytokine and chemokine synthesis via macrophages [60]. Genetic deletion or inhibition of the receptor CCR2 (for a key mediator in the recruitment of inflammatory monocyte chemokine MCP-1) prevented the development of myocardial fibrosis in a model of streptozotocin-induced diabetes [61].

Diabetic myocardial inflammation appears to be mediated through interleukin-1β-dependent pathways, although the genetic depletion of circulating T-cells ameliorated cardiac fibrosis and preserved myocardial contractility in streptozotocin-induced diabetic mice, supporting the role of T-cells in diabetic cardiomyopathy as well [62,63]. A diabetic heart has an increased expression of adhesion molecules and chemokines, which may attract T-cells through mechanisms similar to those involved in myocardial infiltration via macrophages [64]. It is thought the latter that hyperglycemia can activate T-cells through the receptor for advanced glycation end product-dependent pathways, which induce cytokine expression by Th cells [65].

### Study Limitations

The present study has several limitations. The relatively small number of participants might not have provided adequate statistical power, for example, when comparing asymptomatic patients and patients with HFpEF according to monocyte content. Moreover, data for each immune cell subset and some echocardiographic parameters (myocardial work, and diastolic stress test) were not available for every control patient with asymptomatic LVH. That is why the correlations between immune cell count and echocardiographic indices were analyzed only in patients with HFpEF. On the other hand, all asymptomatic patients had grade I LVDD and a normal LV filling pressure, that could make this group homogenous via echocardiographic hemodynamic indices (interindividual variability within normal or insignificantly altered values) and, therefore, could significantly limit the use of correlation analysis in this patient subgroup.

The control group included only patients with asymptomatic LVH and LVDD when myocardial proinflammatory/profibrotic processes had probably been established.

We only analyzed peripheral immune cells, although there may be important differences between circulating and tissue-infiltrating immune cells [66]. CD4^+^ T-cells and monocyte subsets in the bloodstream are not end-differentiated cells and may change their structural affiliation and functional activity in the tissue in the local microenvironment. Accordingly, further studies need to evaluate immune cell subsets in the myocardium and their comparison with blood phenotypes.

The present study had a cross-sectional design, and we could not assess changes in immune cell subsets over time and their relationship to changes in HFpEF clinical and hemodynamic severity, and prognosis, that may be important in better understanding the pathogenesis of HFpEF and in planning appropriate anti-inflammatory immunomodulatory strategies, which need to be investigated in future studies.

## 5. Conclusions

In patients with hypertensive LVH, the clinical deterioration of LVH (transition to HFpEF) and progression of LVDD are probably associated with T-cell imbalance (a decrease in the Treg/Th-act ratio) and an increase in intermediate monocyte count. The more pronounced immune abnormalities were observed in patients with T2DM and HFpEF suggesting the exacerbation of inflammatory processes within the myocardium. These results may form the basis for further focused studies, including investigations of anti-inflammatory/immunomodulatory strategies in HFpEF, which may offer promising approaches for improving prognosis and preventing HFpEF.

## Figures and Tables

**Table 1:** The comparison of patients with LV hypertrophy; asymptomatic vs. HFpEF.

Variables	Asymptomatic LVH (*n* = 21)	LVH + HFpEF (*n* = 36)	*p* Value
*Clinical parameters*			
Age, y	65 (64–74)	68 (60–71)	0.69
Men, *n* (%)	13 (62)	16 (44)	0.21
6 min walk distance, m	485 (434–560)	384 (325–438)	<0.001
NYHA I/II/III, *n* (%)	0/0/0	7/18/11 (19/50/31)	<0.001
Hypertension ^a^, *n* (%)	21 (100)	36 (100)	1.0
Paroxysmal atrial fibrillation, *n* (%)	7 (33)	17 (47)	0.31
Ischemic heart disease, *n* (%)	8 (38)	14 (39)	0.95
Myocardial revascularization, *n* (%)	2 (10)	10 (28)	0.11
Type 2 diabetes mellitus, *n* (%)	5 (24)	16 (44)	0.12
Body mass index, kg/m^2^	29.8 (26.9–31.2)	32.0 (27.4–35.8)	0.15
Obesity ^b^, *n* (%)	10 (48)	23 (64)	0.23
Estimated GFR, mL/min/1.73 m^2^	77 (62–92)	73 (66–88)	0.64
Chronic kidney disease ^c^, *n* (%)	4 (19)	9 (25)	0.61
Systolic BP, mm Hg	135 (130–140)	140 (130–140)	0.88
Diastolic BP, mm Hg	80 (80–90)	85 (80–90)	0.56
Heart rate, bpm	64 (58–68)	65 (61–70)	0.30
NT-proBNP, pg/mL	115 (69–164)	294 (187–583)	<0.001
*Baseline treatments*			
ACEI/ARB, *n* (%)	17 (81)	36 (100)	0.007
β-Blockers, *n* (%)	14 (67)	28 (78)	0.36
Diuretics, *n* (%)	5 (24)	28 (78)	<0.001
Statins, *n* (%)	14 (67)	31 (86)	0.085
Aspirin, *n* (%)	9 (43)	14 (39)	0.77
Mineralocorticoid receptor antagonists, *n* (%)	4 (19)	11 (31)	0.35
SGLT2 inhibitors, *n* (%)	3 (14)	12 (33)	0.12
*Echocardiographic measures*			
LV ejection fraction, %	62 (60–64)	63 (56–66)	0.11
LV GLS, %	19.1 (16.8–20.5)	19.0 (15.8–21.7)	0.71
LV mass index, g/m^2^	114 (102–126)	124 (111–145)	0.033
LA volume index, mL/m^2^	33 (29–42)	42 (37–50)	0.004
LASr, % SR_IVR_, s^–1^ SR_E_, s^–1^	24 (21–26) 0.27 (0.19–0.37) 0.89 (0.80–1.23)	21 (17–24) 0.28 (0.21–0.44) 1.00 (0.73–1.31)	0.046 0.62 0.69
e′, cm/s	7.1 (6.5–8.3)	6.0 (5.1–6.8)	0.002
E/e′ ratio	10.1 (8.4–12.0)	12.5 (10.0–17.3)	0.005
TAPSE, cm	2.5 (2.1–2.7)	2.4 (2.0–2.5)	0.66
PASP, mm Hg	28 (26–33)	39 (31–42)	0.0001
LVDD grade II–III, *n* (%) GWI, mm Hg% GWE, % GCW, mm Hg% GWW, mm Hg%	0 1597 (1377–1750) 89 (84–91) 2029 (1670–2170) 223 (180–287)	18 (50) 1657 (1547–1913) 88 (82–93) 2084 (1924–2279) 265 (167–380)	<0.001 0.29 0.96 0.25 0.42
*Immune cell populations*			
Leucocytes, 10^6^/mL	6.7 (6.2–7.6)	7.4 (5.9–9.1)	0.19
Lymphocytes, 10^6^/mL	1.7 (1.4–2.1)	1.6 (1.2–2.1)	0.33
CD4^+^ T-cells, 10^3^/mL	771 (635–941)	672 (475–882)	0.12
Th-act, 10^3^/mL	296 (248–366)	419 (284–514)	0.016
Treg, 10^3^/mL	34 (27–51)	27 (20–39)	0.054
Treg/Th-act ratio	0.096 (0.089–0.150)	0.074 (0.057–0.087)	0.0009
Monocytes, 10^3^/mL	384 (319–531)	368 (246–536)	0.50
Classical monocytes, 10^3^/mL	291 (271–382)	269 (178–411)	0.50
Non-classical monocytes, 10^3^/mL	62 (52–101)	61 (50–88)	0.58
Intermediate monocytes, 10^3^/mL	23 (19–42)	22 (14–36)	0.35

Data are presented as the median (interquartile range) for continuous variables, and as percentages for categorical variables. ^a^—blood pressure, ≥140/90 Hg mm; ^b^—body mass index, ≥25 kg/m^2^; ^c^—eGFR, <60 mL/min/1.73 m^2^. ACEI, angiotensin-converting enzyme inhibitor; ARB, angiotensin receptor blocker; BP, blood pressure; E, early inflow velocity; e′, averaged annulus relaxation velocity; eGFR, estimated glomerular filtration rate; GLS, global longitudinal strain; GWE, global myocardial work efficiency; GWI, global myocardial work index; GLS, global longitudinal strain; GCW, global constructive work; GWW, global wasted work; HFpEF, heart failure with a preserved ejection fraction; LASr, left atrial strain during the reservoir phase; LVDD, left ventricular diastolic dysfunction; LVH, left ventricular hypertrophy; NT-proBNP, N-terminal pro-brain natriuretic peptide; PASP, pulmonary artery systolic pressure; SGLT2, sodium–glucose transport protein 2; TAPSE, tricuspid annular plane systolic excursion; Th-act, activated T-helper cells; Treg, regulatory T-cells.

**Table 2:** Diagnostic accuracy of echocardiographic variables and immune cell counts in revealing HFpEF among patients with LVH.

Variable	AUC (95% CI)	*p* Value	Comparison of AUC (95% CI)	*p* Value for Comparison of AUC	Sensitivity (%)	Specificity (%)
NT-proBNP (>200 pg/mL)	0.86 (0.72–0.95)	<0.0001	0.02 (−0.16 to 0.19)	0.86	74	85
Treg/Th-act ratio (≤0.080)	0.82 (0.79–0.92)	<0.0001	Reference	–	70	92
PASP (>36 mm Hg)	0.82 (0.69–0.91)	<0.0001	−0.02 (−0.20 to 0.13)	0.73	61	95
e′ (≤6.9 cm/s)	0.75 (0.61–0.85)	0.001	−0.08 (−0.36 to 0.07)	0.20	83	65
LA volume index (>38 mL/m^2^)	0.73 (0.60–0.84)	0.0002	−0.05 (−0.13 to 0.23)	0.61	74	71
E/e′ ratio (>9.5)	0.70 (0.56–0.82)	0.005	−0.12 (−0.38 to 0.05)	0.13	83	50
Th-act (>371 × 10^3^/mL)	0,70 (0.55–0.82)	0.021	−0.13 (−0.35 to 0.08)	0.22	58	92
LV mass index (>136 g/m^2^)	0.67 (0.53–0.79)	0.019	−0.18 (−0.36 to −0.02)	0.033	33	95
Treg (≤22.3 × 10^3^/mL)	0.66 (0.52–0.79)	0.039	−0.17 (−0.37 to −0.03)	0.02	39	94
LASr (≤20.0%)	0.65 (0.51–0.77)	0.04	−0.21 (−0.42 to 0.0)	0.047	46	85

AUC, area under curve; CI, confidence interval. Other abbreviations are the same as those in Table 1.

**Table 3:** Correlation of total monocytes and their subsets with echocardiographic parameters ^1^.

Variable	Intermediate Monocytes		Classic Monocytes		Non-Classic Monocytes	
	r	*p* Value	r	*p* Value	r	*p* Value
LV ejection fraction	−0.34	0.049	−	−	−	−
LV GLS during rest	−0.50	0.003	−	−	−	−
Change in LV GLS during exercise	−0.35	0.045	−	−	−	−
Global myocardial work index	−0.57	0.0008	−	−	−	−
Global myocardial work efficiency	−0.44	0.013	−	–	–	–
Global constructive work	−0.52	0.003	−	–	–	–
Global wasted work	–	–	0.43	0.015	0.41	0.023
TAPSE during rest	−0.45	0.006	–	–	–	–
Change in TAPSE during exercise	–	–	−0.34	0.049	−0.36	0.035
Mitral annular e′ during rest	−0.34	0.047			–	–
Change in e′ during exercise	–	–	−0.36	0.034	–	–
Mitral E/e′ ratio during rest	0.45	0.039	–	–	–	–
Pulmonary vein S/D ratio	−0.36	0.035	–	–	–	–
LASr	−0.40	0.020	–	–	–	–
SR_IVR_	−0.53	0.002	–	–	–	–
SR_E_	−0.35	0.040	–	–	–	–
RV wall thickness	–	–	0.35	0.043	–	–
Tricuspid E/A ratio	–	–	0.46	0.007	–	–
Tricuspid E/e′ ratio	0.43	0.012	–	–	–	–
LV mass index	–	–	–	–	–	–

^1^—only significant correlations are presented. r, correlation coefficient; RV, right ventricular; S, systole; SR_E_, global strain rates during early diastole; SR_IVR_, global strain rate during the isovolumic relaxation period. Other abbreviations as in Table 1.

**Table 4:** The comparison of patients with HFpEF depending on diabetes status.

Variables	HFpEF with Diabetes (*n* = 16)	HFpEF without Diabetes (*n* = 20)	*p* Value
*Clinical parameters*			
Age, y	70 (66–72)	66 (56–70)	0.21
Men, *n* (%)	5 (31)	11 (55)	0.16
6 min walk distance, m	363 (319–420)	424 (363–473)	0.021
NYHA I/II/III, *n* (%)	1/6/9 (6/38/56)	6/12/2 (30/60/10)	0.008
Body mass index, kg/m^2^	35.5 (29.8–37.6)	30.3 (26.8–32.8)	0.045
Estimated GFR, mL/min/1.73 m^2^	73 (58–83)	77 (68–91)	0.15
NT-proBNP, pg/mL	339 (224–623)	212 (116–373)	0.065
*Echocardiographic measures*	2 (10)	10 (28)	0.11
LV ejection fraction, %	62 (56–65)	64 (57–67)	0.65
LV GLS, %	18.7 (14.9–20.6)	19.0 (16.7–22.0)	0.68
LV mass index, g/m^2^	123 (109–140)	124 (114–149)	0.42
LA volume index, mL/m^2^	45 (38–52)	41 (36–48)	0.69
LASr, %	19 (15–22)	22 (20–26)	0.064
E/e′ ratio	13.5 (11.4–17.3)	11.8 (9.4–16.0)	0.049
TAPSE, cm	2.1 (1.8–2.4)	2.5 (2.2–2.6)	0.015
PASP, mm Hg	40 (32–43)	37 (30–40)	0.52
LV diastolic dysfunction grade II–III, *n* (%)	8 (50)	10 (50)	1.0
*Immune/inflammatory cell populations*			
Leucocytes, 10^6^/mL	7.4 (5.9–9.8)	7.5 (5.9–9.0)	0.46
Lymphocytes, 10^6^/mL	1.5 (1.2–2.1)	1.7 (1.3–2.2)	0.47
CD4^+^ T-cells, 10^3^/mL	672 (450–802)	658 (498–902)	0.37
Th-act, 10^3^/mL	426 (271–514)	411 (319–519)	0.69
Treg, 10^3^/mL	22 (14–38)	29 (23–40)	0.089
Treg/Th-act ratio	0.068 (0.050–0.074)	0.079 (0.064–1.00)	0.036
Monocytes, 10^3^/mL	399 (228–513)	355 (252–546)	0.91
Classical monocytes, 10^3^/mL	269 (167–398)	275 (190–420)	0.81
Non-classical monocytes, 10^3^/mL	77 (48–93)	58 (50–75)	0.25
Intermediate monocytes, 10^3^/mL	28 (15–44)	17 (13–25)	0.049

Data are presented as the median (interquartile range) for continuous variables, and as percentages for categorical variables. All abbreviations are the same as those in Table 1.

## Data Availability

The authors confirm that the data supporting the findings of this study are available within the article. Raw data that support the findings of this study are available from the corresponding author, upon reasonable request.
